# The Effects and Mechanisms of pH and Dissolved Oxygen Conditions on the Release of Arsenic at the Sediment–Water Interface in Taihu Lake

**DOI:** 10.3390/toxics11110890

**Published:** 2023-10-30

**Authors:** Liqing Zeng, Changzhou Yan, Fan Yang, Zhuo Zhen, Jiaming Yang, Jielun Chen, Yujie Huang, Yuhui Xiao, Wen Zhang

**Affiliations:** 1 Department of Public Health and Medical Technology, Xiamen Medical College, Xiamen 361021, China; zlq@xmmc.edu.cn (L.Z.); 202106520052@xmmc.edu.cn (J.Y.); 202106520061@xmmc.edu.cn (J.C.); 202106520065@xmmc.edu.cn (Y.H.); 202106520051@xmmc.edu.cn (Y.X.); 202106520055@xmmc.edu.cn (W.Z.); 2 Key Laboratory of Urban Environment and Health, Institute of Urban Environment, Chinese Academy of Sciences, Xiamen 361021, China; fyang@iue.ac.cn (F.Y.); zhuozhen@iue.ac.cn (Z.Z.)

**Keywords:** pH, dissolved oxygen, sediment–water interface, Taihu Lake

## Abstract

The pH and dissolved oxygen (DO) conditions are important environmental factors that control the migration of arsenic (As) at the sediment–water interface. This study investigates the distribution differences of reactive iron, manganese, and arsenic at the sediment–water interface under anaerobic and aerobic conditions at different pH levels. The strong buffering capacity of sediment to water pH results in a shift towards neutral pH values in the overlying water under different initial pH conditions. The level of DO becomes a key factor in the release of As from sediment, with lower DO environments exhibiting higher release quantities and rates of As compared to high DO environments. Under low DO conditions, the combined effects of ion exchange and anaerobic reduction lead to the most significant release of As, particularly under pH 9.5 conditions. The formation of amorphous ferrous sulfide compounds under low DO conditions is a significant factor contributing to increased arsenic concentration in the interstitial water. Therefore, the re-migration of endogenous arsenic in shallow lake sediments should consider the combined effects of multiple driving forces.

## 1. Introduction

Arsenic is a non-essential element of the human body and is widely recognized as a potent carcinogen. Its carcinogenic risk is much higher than other traditional pollutants [1]. Generally, naturally occurring arsenate has low solubility and does not pose significant harm to humans and ecosystems. However, in recent decades, with the rapid development of industry and agriculture, activities such as mining, smelting, production of glass and electronic products, waste disposal, use of pesticides and herbicides, and the use of animal feed or combustion of fossil fuels have resulted in large amounts of arsenic pollutants entering freshwater systems and accumulating in sediments through processes such as adsorption, settling, or coagulation [2].

Shallow lakes, as typical freshwater systems, exhibit complexity, diversity, and rapid physical, chemical, and biological processes. The exchange of substances between the water and sediment in shallow lakes is frequent, and changes in the aquatic environment can easily lead to changes in sediment environments. Sediments, as the main adsorbents for pollutants, can potentially release pollutants back into the water and lead to their diffusion [3,4], thereby affecting water quality and safety. Therefore, the endogenous release of pollutants from sediments in shallow lakes has received great attention from scholars both domestically and internationally [5,6]. Arsenic, as a pollutant sensitive to environmental conditions, is affected by changes in redox conditions, pH, temperature, and organic matter, among other factors. The main forms of arsenic in sediments are As(V) and As(III), and their transformation and release are influenced by dissolution, ion exchange, desorption, and redox reactions. These processes are closely related to the anaerobic environment of sediments and corresponding acidity or alkalinity [7,8], with pH and dissolved oxygen (DO) being recognized as key factors that control arsenic migration at the sediment–water interface. In recent years, the increasing rate of total arsenic concentration in lake waters has attracted public attention [9]. Therefore, studying the characteristics of arsenic release at the sediment–water interface in shallow lakes and its influencing factors is of significant academic value and practical importance.

The pH and dissolved oxygen (DO) in the environment play a role in the transformation of arsenic species in sediments. Under near-neutral or acidic conditions, negatively charged H_2_AsO_4_^−^ and HAsO_4_^2−^ are the main forms of arsenic in aerobic sediments and overlying water [10]. These forms can be effectively immobilized by positively charged substances such as hydrated metal (hydro)oxides of iron, aluminum, and manganese, colloids, and clay minerals through processes like adsorption, co-precipitation, or surface complexation reactions [11]. Under oxidizing conditions, more reactive trivalent arsenic can be oxidized to less reactive pentavalent arsenic, which can be re-adsorbed or (co-)precipitated with various mineral phases [12]. Aerobic decomposition of organic matter in sediments can also convert some organic arsenic to inorganic arsenic, which is an important source of arsenic release under oxidizing conditions [13]. Under aerobic conditions in the water column, when sediment pH increases, trivalent and pentavalent arsenic exist in forms such as H_3_AsO_3_, H_2_AsO_3_^−^, HAsO_4_^2−^, and AsO_4_^3−^, which are less prone to adsorption, precipitation, or complexation by negatively charged hydroxylated iron [14]. Therefore, the desorption capacity of arsenic is higher in aerobic alkaline environments compared to acidic conditions. Additionally, increasing OH^−^ concentrations can compete with arsenate for adsorption sites on other carriers, reducing the adsorption capacity of these carriers and further increasing the concentration of dissolved arsenic [15]. However, some studies have shown that under aerobic conditions, with an increase in pH, the formation of iron and manganese (hydro)oxides in sediments strengthens the adsorption of arsenic by these minerals, leading to an increase in the association of arsenic with iron and manganese (hydro)oxides as pH increases [16]. Under reducing conditions, an increase in pH can also promote the reduction in arsenic to less dissociated arsenous acid, leading to a further increase in the exchangeable arsenic content in sediments and an increase in its biological toxicity [17]. The release of arsenic from sediments due to reduction reactions under anaerobic conditions is much stronger than the release caused by the oxidation of organic arsenic under aerobic conditions. However, in water bodies with severe organic pollution, aerobic conditions can actually lead to the rapid release of arsenic from sediments, as the mineralization of organic matter under aerobic conditions releases dissolved arsenic at a much faster rate than under anaerobic conditions [18,19,20].

In shallow lakes, the dissolved oxygen (DO) concentration typically affects the redox potential (Eh), and changes in Eh have significant impacts on the transformation of iron/manganese (hydro)oxides, the conversion between pentavalent arsenic and trivalent arsenic, sulfur transformation, and microbial activities. These factors consequently influence the morphological changes and migration capabilities of arsenic in sediments [21]. Previous studies have investigated the influence of pH and Eh on arsenic migration in shallow lake sediments using fixed values of pH and Eh [22]. Some studies have focused only on the effects of pH or Eh variations on arsenic migration [23,24]. However, there is a relatively limited understanding of the impact of pH and DO variations during the cultivation process on arsenic release in shallow lake sediments, especially regarding the changes in different arsenic species. The specific processes of these changes are not yet fully understood.

Lake Taihu is the largest shallow lake and the second-largest freshwater lake in China, making vital contributions to freshwater supply, agricultural irrigation, water resources development, and navigation in the Yangtze River Delta region [25]. This experiment aims to analyze the release changes of arsenic at the sediment–water interface of Lake Taihu sediments under different DO and initial pH conditions and investigate the factors related to arsenic migration in different conditions. The goal is to understand the differences in arsenic release behavior in shallow lakes under different DO and initial pH conditions and clarify the relationship between arsenic migration and changes in different arsenic components in sediments. This research provides the corresponding technical and theoretical support for the prevention and control of arsenic pollution in shallow lakes.

## 2. Materials and Methods

### 2.1. Sample Collection and Preparation

In January 2016, samples were collected from Zhushan Bay in Lake Taihu (31°22′53″ N, 120°03′11.5″ E) for indoor simulated experiments. Water samples were collected below a depth of 0.50 m using a water sampler, while surface sediment samples of around 10 cm were collected using a grab sampler. The sediment samples were thoroughly mixed and stored in clean polyethylene plastic bags, with air excluded, at a low temperature and in the dark. They were transported to the laboratory as soon as possible. The sediment samples were sieved through a 1.0 mm mesh sieve to remove shellfish and gravel debris, and then thoroughly mixed for use in indoor simulated experiments. Some of the sediment samples were freeze-dried, ground to pass through a 100-mesh nylon sieve, and used for the analysis of the basic physicochemical properties of the sediment and the continuous extraction of As chemical components.

### 2.2. Experimental Mesocosms

Under ambient temperature conditions, fresh sediment was uniformly added to transparent Plexiglas tubes (Φ × L: 7 cm × 50 cm) to a height of 12 cm, and the bottoms of the tubes were sealed with rubber stoppers. To prevent the sediment from re-suspending, water was slowly added along the tube wall until the water column reached a height of 30 cm. All tubes were wrapped in aluminum foil to avoid light exposure. Two replicates were set for each experimental condition. The complete glass tubes were placed in plastic boxes filled with lake water to a height of 42 cm. After a two-week equilibration period, a new sediment–water interface was formed. Based on the acidity changes in Lake Taihu in recent years, the pH of the overlying water was adjusted to 6.2, 7.5, and 9.5 using 1 mol/L HCl and NaOH, respectively.

The top of each tube was sealed with a rubber stopper equipped with a venting tube and an exhaust tube. One end of the venting tube was connected to a gas cylinder, and the other end was immersed in the water column. The height of the venting tube was adjusted to minimize disturbance to the sediment. The air or N_2_ flow rate and duration entering the overlying water were adjusted to create high and low dissolved oxygen (DO) conditions. In the high DO group, an air pump was used for continuous aeration, and the oxygen flow rate was controlled using a spiral clamp to maintain the dissolved oxygen concentration in the experimental system between 5 mg/L < DO < 7 mg/L. In the low DO group, pure N_2_ with a purity of 99.9% was introduced into the system once in the morning and once in the evening during the cultivation period, with each introduction lasting 1 h. After the ventilation was completed, the exhaust tube was sealed to ensure that the DO in the experimental group was <1 mg/L. All tubes were kept still at 25 °C. The experiment lasted 30 days. Sampling was carried out on days 1, 2, 6, 17, and 30 during the cultivation period. The collected water samples were filtered through 0.45 μm cellulose acetate membranes, and the sediment samples were sub-sampled at 2 cm intervals. The interstitial water was collected by filtering the centrifuged sediment samples at 5000 rpm for 15 min through 0.45 μm cellulose acetate membranes. The collected samples were stored at −20 °C for elemental analysis. The changes in the pH and Eh of the overlying water and sediment were continuously monitored throughout the experiment. The water level of the overlying water was maintained, and if any loss occurred due to evaporation or other reasons, it was replenished with lake water.

### 2.3. Sampling and Analysis

Indoor overlying water was analyzed using a multi-parameter water quality analyzer (Multi 3420, WTW, Munich, Germany). The pH of the sediment was determined by preparing a suspension of sediment and deionized water (CO_2_-free) in a ratio of 1:2.5. The suspension was shaken for 30 min at 250 rpm and then centrifuged at 4000 rpm for 10 min. The pH of the supernatant was measured using a pH meter. The moisture content was determined by weighing approximately 10 g of wet sediment, drying it in an oven at 105 ± 3 °C for 12 h, allowing it to cool in a desiccator for 30 min, and repeating this drying process until a constant weight was achieved. The difference in weight was calculated [26]. The pH and Eh values of the sediment profile were determined using microelectrodes (Unisense, Aarhus, Denmark). The total organic carbon (TOC) content was also determined using a TOC analyzer (TOC-VCPH, Elementar, Frankfurt, Germany).

For the analysis of elemental concentrations in the water phase, including overlying water and interstitial water, the total dissolved arsenic and manganese contents were measured using an Inductively Coupled Plasma Mass Spectrometer (ICP-MS, Agilent7500cx, Santa Clara, CA, USA) in helium mode to exclude possible interference from ^40^Ar and ^35^Cl [27]. Internal standards with ^115^In and ^103^Rh were used to assess the stability of the ICP-MS instrument. The iron content in the water phase was determined using the ortho-phenanthroline colorimetric method [28], and the sulfur content was determined using the methylene blue method [29].

For speciation analysis of elements, arsenic species (As(III) and As(V)) were determined using a High Performance Liquid Chromatography (HPLC, Agilent1200, Santa Clara, CA, USA) coupled with an ICP-MS (AgilentLC1100, Santa Clara, CA, USA), following the methods described by Zhu et al. and Zhang et al. [30,31]. An anion exchange column (PRP-X100, 250 × 4.1 mm, Hamilton) with a guard column (11.2 mm, 12–20 μm) was used. The mobile phase was prepared by mixing 10 mmol/L NH_4_H_2_PO_4_ (chromatographically pure) and 10 mmol/L NH_4_NO_3_ (optically pure), adjusting the pH to 6.2 with HNO_3_ or ammonia solution (optically pure). After passing through the membrane, the mobile phase was used to elute and separate arsenic species at a flow rate of 1.0 mL/min, with a ratio of 75% mobile phase and 25% ultra-pure water.

For sediment analysis, the total concentrations of arsenic, manganese, and iron were determined using an X-ray Fluorescence Spectrometer (XRF, AxiosmAx; PANalytical, Almelo, The Netherlands). The elemental chemical forms in the sediment were analyzed using Wang et al.’s proposed method [32], which involves a sequential extraction of water-soluble arsenic (F1), exchangeable arsenic (F2), amorphous iron-bound arsenic (F3), crystalline iron-bound arsenic (F4), organic-bound arsenic (F5), and residual arsenic (F6). The sum of the iron and manganese contents extracted simultaneously from F3 and F4 was defined as FeOOH and MnOOH.

### 2.4. Quality Assurance and Data Analyses

To ensure the feasibility and precision of the sample analysis method, the standard sediment sample GSD-9 was used as a reference for the determination of total elemental concentrations in sediment and the extraction of arsenic fractions. The total arsenic and manganese concentrations in the water were controlled using arsenic standard substance (GSB 07-1714-2004) and manganese standard substance (GSB 04-1736-2004), respectively. The standard control for iron and sulfur in the water phase was prepared using ammonium ferrous sulfate hexahydrate and sodium sulfide. The arsenic species As(V) and As(III) were controlled using standard substances prepared from Na_3_AsO_4_∙12H_2_O (Fluka) and NaAsO_2_ (Alfa Aesar), respectively. All stock solutions of elements were stored at −20 °C and thawed and diluted to obtain the required standard curves before measurement. All reagents used in the experiment were of analytical grade (National Pharmaceutical Group Chemical Reagent Co., Ltd., Shanghai, China) and were prepared using ultrapure water. The recovery rate of the standard was 95–106% (n = 4), with a relative standard deviation within ±5%. Three replicates were performed for each sample measurement in order to ensure an analysis relative error of less than 10%. Glassware and centrifuge tubes were soaked in 10% HNO_3_ (optically pure) for at least 48 h before use and rinsed with ultrapure water.

The formula for the cumulative release rate of each element is as follows:

(1) $$r=\frac{0.075V(C_{n}-C_{0})}{(t_{n}-t_{0})A}$$

r (mg/(m^2^∙d)) represents the cumulative release rate of the element, C_n_ (mg/L) is the concentration of the element in the overlying water on the nth day, C_0_ (mg/L) is the initial concentration of the element in the overlying water, A (m^2^) represents the surface area of the sediment-water contact, and V (L) represents the volume of the overlying water. The time points t_n_ and t_0_ represent the nth day of cultivation and the initial time, respectively.

## 3. Results and Discussions

### 3.1. Basic Physicochemical Properties of Sediments

The basic physicochemical properties of the surface sediments collected from Zhushan Bay in Taihu Lake are shown in Table 1. The pH value of the sediments was 7.56, indicating a neutral environment. The organic matter deposition in this lake area is significant, with a TOC (total organic carbon) content of 1.44%. FeOOH and MnOOH were important forms of iron and manganese in Taihu Lake sediments.

### 3.2. Effects of Initial pH of Water on Physicochemical Properties of Sediments under Different DO Conditions

**Figure 1:**
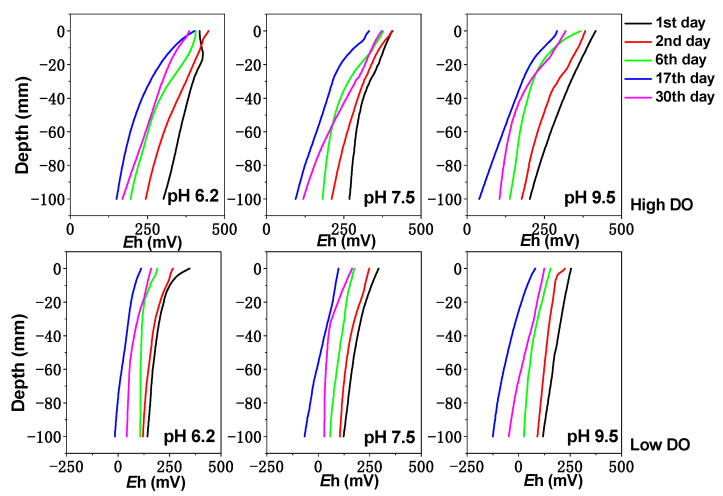
The effect of initial pH of water body on sediment *E*h under different DO conditions.

**Figure 2:**
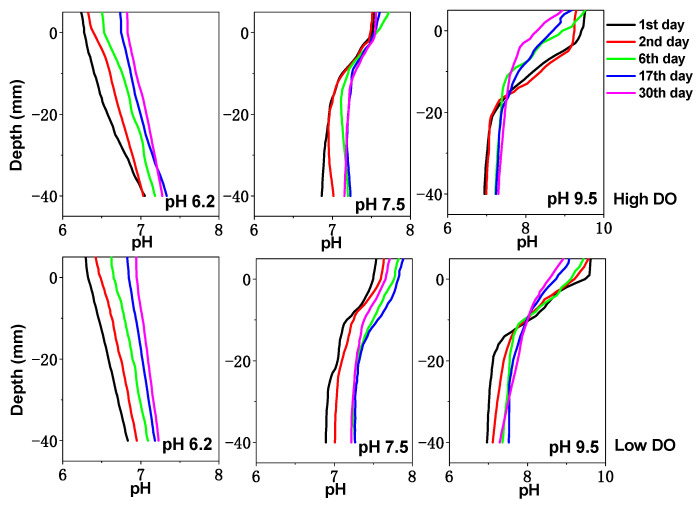
The effect of initial pH of water body on sediment’s pH under different DO conditions.

The pH and Eh value changes of the sediment are shown in Figure 1 and Figure 2. Under high and low DO conditions, the Eh value of the sediment shows a trend of initially decreasing and then increasing. Under initial pH 9.5 conditions, the potential decrease was the most significant. In the early stage of high DO cultivation, the difference in the sediment Eh was not significant between different pH conditions; then, the sediment Eh under initial pH 6.2, 7.5, and 9.5 conditions decreased to ranges of +149 to +386 mV, +118 to +372 mV, and +105 to +319 mV, respectively, at the end of cultivation. However, under low DO cultivation, the sediment Eh under initial pH 6.2, 7.5, and 9.5 conditions decreased to ranges of −16 to +112 mV, −67 to +99 mV, and −125 to +81 mV on day 17 and then slowly increased. This phenomenon can be attributed to the consumption of organic matter in sediments by microorganisms whose activity was then inhibited [33], leading to a gradual increase in the redox potential by day 30. Therefore, both the initial pH and DO have a significant impact on sediment Eh. From Figure 2, it can be seen that under initial pH 6.2, 7.5, and 9.5 conditions in the early stage of cultivation (0–6 days), the pH values at the sediment–water interface (0 to −2 cm) were greatly influenced by the initial pH of the overlying water. With the increase in cultivation time, the pH at the sediment–water interface changed due to the buffering effect of the sediment. After cultivation, the pH at the sediment–water interface under initial pH 6.2 and 9.5 conditions varied within ranges of 6.83–7.09 and 7.47–8.71, respectively, while under initial pH 7.5 conditions, the pH value at the sediment–water interface was similar to the initial pH value, ranging from 7.21 to 7.66. Compared to high DO conditions, the pH value at the sediment–water interface was slightly larger during low DO cultivation, which might be more favorable for proton consumption under low DO reducing conditions. Therefore, the initial pH and DO of the water had a certain influence on sediment pH.

### 3.3. Effect of Initial pH on Dissolved Fe, Mn and S under Different DO Conditions

**Figure 3:**
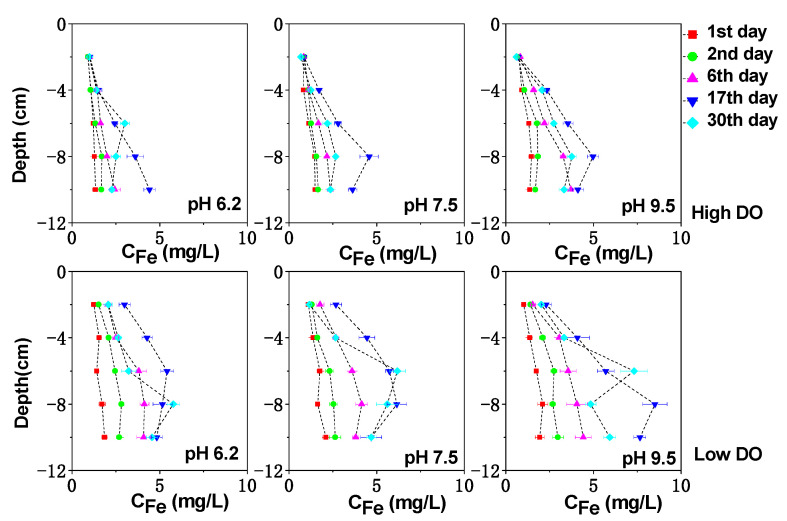
The effect of initial pH of water on dissolved Fe under different DO conditions.

**Figure 4:**
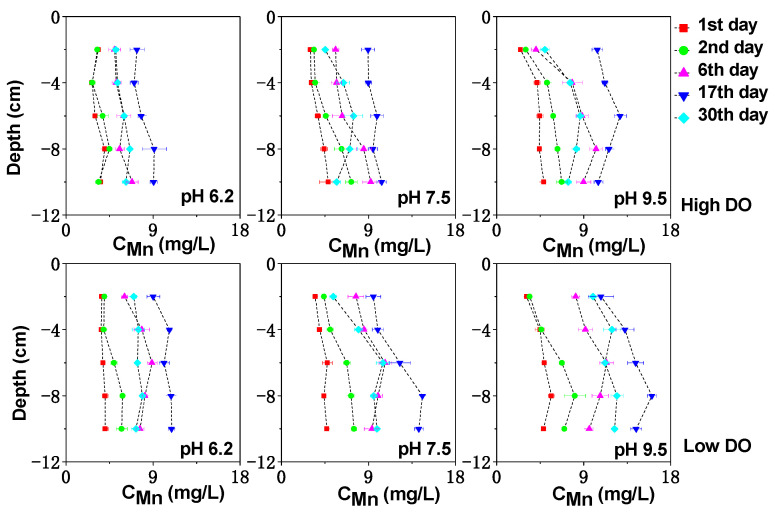
The effect of initial pH of water on dissolved Mn under different DO conditions.

**Figure 5:**
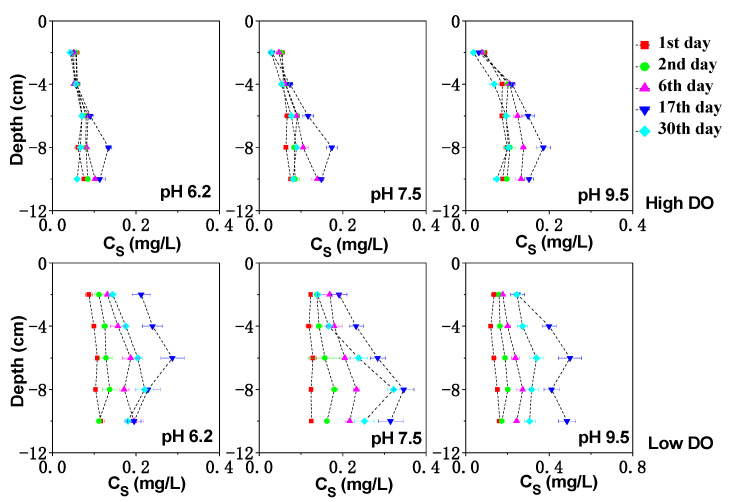
The effect of initial pH of water on dissolved S under different DO conditions.

The vertical distribution of dissolved Fe, Mn, and S in the sediment can be seen in Figure 3, Figure 4 and Figure 5. Under high DO conditions, the profiles of dissolved Fe and Mn were similar. In the initial stage of cultivation (0–6 days), the concentrations near the sediment–water interface (0–2 cm) were low, and the peaks mainly occurred in the deeper sediment. Especially at an initial pH of 9.5, the concentrations of dissolved Fe and Mn near the sediment–water interface were the lowest compared to other initial pH conditions, ranging from 0.76 to 0.82 mg/L and 2.47 to 4.07 mg/L, respectively, as both Fe and Mn could be oxidized and precipitated easily under strong alkaline conditions. As the cultivation progresses, the sediment pH changed to neutral or weak alkaline conditions. Due to the abundant presence of easily biodegradable organic matter in the sediment of Zhushan Bay in Lake Taihu [34], strong mineralization occurred and the deep sediment was in a weak reducing environment (+100 mV–+200 mV) (Figure 1). The reduction in Fe/Mn-OOH intensified, and the concentrations of dissolved Fe and Mn further increased and gradually migrated to the upper layer (Figure 3).

The average concentration of dissolved Mn in the interstitial water increased continuously until the 17th day to 8.07 mg/L (pH 6.2), 9.53 mg/L (pH 7.5), and 11.31 mg/L (pH 9.5), which was an increase of 1.43 times, 1.50 times, and 1.77 times compared to the initial concentration. Similarly, the average concentration of dissolved Fe in the interstitial water increased to 2.36 mg/L (pH 6.2), 2.24 mg/L (pH 7.5), and 2.71 mg/L (pH 9.5), corresponding to increases of 1.24 times, 1.37 times, and 1.71 times compared to the initial concentration. After cultivation for 30 days, the Eh of the sediment increased under different pH conditions, leading to the oxidation and adsorption precipitation of dissolved Fe and Mn, resulting in a decrease in their concentrations. Due to the higher redox potential of Mn compared to Fe [34], MnOOH acted as the oxidant for the decomposition of organic matter in the sediment and was reduced preferentially, while the rapid desorption of exchangeable manganese minerals in the sediment also contributed to the rapid increase in dissolved Mn concentration [35]. These factors led to the rapid release of dissolved Mn in the interstitial water, and a peak concentration was observed at shallower depths (Figure 4).

The concentration of dissolved sulfur in the interstitial water remained relatively stable or even decreased slightly, and a significant increase in the concentration of dissolved sulfur in the interstitial water was observed in the deep sediment on the 17th day (Figure 5). The average concentration of dissolved sulfur in the interstitial water varied within the range of 0.059–0.088 mg/L (pH 6.2), 0.064–0.011 mg/L (pH 7.5), and 0.072–0.13 mg/L (pH 9.5).

In continuous cultivation under low dissolved oxygen (DO) conditions, the release intensity of dissolved Fe and Mn in the interstitial water increased as the pH decreases. Similar to high DO cultivation, under initial pH conditions of 6.2, 7.5, and 9.5, the concentration of dissolved Fe (Mn) in the interstitial water continued to increase until day 17, reaching 4.54 mg/L (10.33 mg/L), 4.74 mg/L (12.10 mg/L), and 5.65 mg/L (13.79 mg/L), respectively. By day 30, the sediments in all treatment groups remained in a moderately reduced environment (Figure 1), and the concentration of dissolved Fe and Mn in the interstitial water decreased due to the formation of secondary minerals such as iron oxides, CO_3_^2−^, and S^2−^ [36,37,38] (Figure 3 and Figure 4). Comparatively, under the initial pH 9.5 condition, the Eh of the sediments remained at a lower level (Figure 1), indicating a higher reduction capacity for Fe/Mn-OOH. Therefore, higher concentrations of dissolved Fe and Mn accumulated in the interstitial water, especially in the deep sediment layers where the concentrations were significantly higher compared to the other initial pH conditions (Figure 3 and Figure 4) (*p* < 0.05).

The production of dissolved S was closely related to microorganisms. For example, sulfate-reducing bacteria could convert sulfate into sulfide [39]. In a highly organic-rich reducing environment, the reduction behavior of sulfate-reducing bacteria was enhanced [40]. Under the initial pH 9.5 condition, the microbial activity at the sediment–water interface could be inhibited due to the higher initial pH of the water, which was unfavorable for the generation of dissolved S. As a result, the concentration of dissolved S at the sediment–water interface (0–−2 cm) only varied within the range of 0.14–0.18 mg/L (Figure 5). As the buffer capacity of the sediment increased, the pH at the sediment–water interface gradually decreased to a weakly alkaline range (Figure 2), allowing the microbial activity to recover gradually and facilitating the accelerated reduction in sulfate in anaerobic conditions. Starting from day 17, the concentration of dissolved S at the sediment–water interface rapidly increased to 0.25 mg/L. The average maximum concentrations of dissolved S under different pH conditions were 0.23 mg/L (pH 6.2), 0.27 mg/L (pH 7.5), and 0.41 mg/L (pH 9.5), which was significantly higher than the maximum concentrations under corresponding conditions in high DO cultivation (Figure 5). By day 30, the concentrations of dissolved S in all treatment groups no longer increased but instead decreased, which might be related to the precipitation of sulfides under reducing conditions [41].

**Figure 6:**
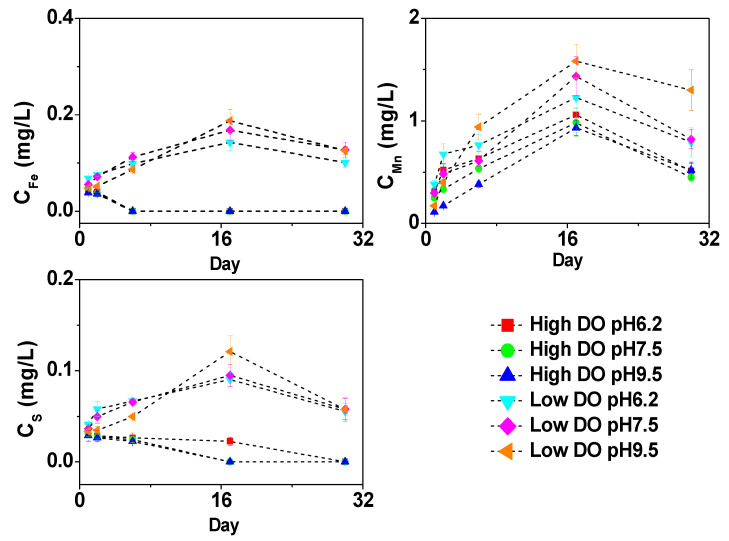
The effect of initial pH of water body on Fe, Mn, and S concentration of overlying water under different DO conditions.

During the cultivation process, changes in the dissolved forms of Fe, Mn, and S in the overlying water also occurred (Figure 6). In a high dissolved oxygen (DO) environment, dissolved oxygen has a greater inhibitory effect on the oxidation of dissolved Fe and S in the water. The concentration of dissolved Fe in the overlying water gradually decreased until it was below the detection limit, and the concentration of dissolved S also decreased gradually. Mn(II) usually undergoes oxidation under conditions with a pH ≥ 9 [42], while the overlying water in the cultivation process remained within a neutral to weakly alkaline range (pH < 9), which is not conducive to the oxidation of Mn. Therefore, dissolved Mn can still be detected in the overlying water. During the first 6 days of cultivation, there were significant differences in the concentration of dissolved Mn in the overlying water under different pH conditions (*p* < 0.05). Subsequently, as the water pH changed to neutral or weakly alkaline, there were no significant differences in the concentration of dissolved Mn in the overlying water under different pH conditions. On the 30th day, the concentration of dissolved Mn in the overlying water was 0.51 mg/L (pH 6.2), 0.45 mg/L (pH 7.5), and 0.52 mg/L (pH 9.5).

In a low DO environment, the concentration changes in dissolved Fe, Mn, and S in the overlying water under different pH conditions were similar to those of the corresponding elements in the pore water, increasing continuously until day 17 and then decreasing. The concentrations of dissolved Fe, Mn, and S in the overlying water under different pH conditions were significantly higher than those in the high DO environment (Figure 6). In the initial stage of cultivation under low DO and pH 6.2, the pH of the overlying water was weakly acidic (Figure 6). The release of dissolved Fe and Mn at the sediment–water interface was mainly attributed to the reduction in the surface sediment Fe/Mn-OOH, but there was also a partial contribution from the acidic dissolution of Fe/Mn-OOH, leading to a relatively high release intensity. Meanwhile, under acidic conditions, sulfate generated from reduction was more likely to exist in the dissolved form [43], resulting in significantly higher concentrations of dissolved Fe, Mn, and S in the overlying water under the initial pH 6.2 condition (Figure 6).

As the water pH buffered to a weakly neutral range, the acidic dissolution of Fe/Mn-OOH at the sediment–water interface decreased. Due to the relatively high Eh of the system (Figure 1), the reduction intensity of Fe/Mn-OOH and sulfate was lower, resulting in lower concentrations of dissolved Fe, Mn, and S transferred to the overlying water. In the initial stage of cultivation under low DO and pH 9.5, the high pH was unfavorable for the existence of dissolved Fe, Mn, and S, resulting in low concentrations. As the water pH shifted to weakly alkaline conditions under the influence of low DO, the concentrations of dissolved Fe, Mn, and S in the overlying water sharply increased to 0.19 mg/L, 1.58 mg/L, and 0.12 mg/L on day 17, followed by a slight decrease.

### 3.4. Effect of Initial pH on Dissolved As in Interstitial Water under Different DO Conditions

In the initial stage of cultivation under high DO and pH 6.2, the sediment–water interface (0 to −2 cm) exhibited a weakly acidic environment (Figure 2), resulting in a positively charged surface of the sediment that can adsorb arsenate anions from the water and inhibit the dissociation of arsenate, thus reducing the release potential of dissolved As. However, the weakly acidic conditions also favored the acid dissolution of Fe/Mn-OOH, which served as the preferential adsorption medium for the sediment As. Hence, some of the As released from the upper sediment during the first 0–6 days of cultivation could be attributed to the acidic dissolution of Fe/Mn-OOH. Additionally, at this stage, the higher electrical potential (~+400 mV) at the sediment–water interface not only promoted the reduction in MnOOH (Lovley, 1991) but also facilitated the oxidation of arsenic sulfides to some extent [44]. These factors may collectively contribute to the increased concentration of dissolved As(V) + As(III) at the sediment–water interface on the 6th day, reaching 9.95 μg/L.

By the 17th day of cultivation, the upper sediment gradually transitioned to weakly neutral conditions, leading to a decreased acid dissolution of Fe/Mn-OOH at the sediment–water interface. Furthermore, due to the mineralization of sediment organic matter, the redox potential (Eh) at the sediment–water interface decreased, which was unfavorable for the oxidation of arsenic sulfides. As a result, the reduction in Mn-OOH became the main source of arsenic release at the sediment–water interface, while Fe-OOH exhibited less stability and showed lower affinity for dissolved As under weakly neutral conditions. Consequently, the concentration of dissolved As(V) + As(III) at the sediment–water interface continued to increase, reaching 14.69 μg/L, with an As(III)/As(V) ratio of 0.85.

By the 30th day of cultivation, the increase in Eh led to a simultaneous decrease in the concentrations of dissolved As(V) + As(III) and dissolved Fe and Mn in the pore water, indicating potential co-precipitation and co-adsorption processes. Overall, under high DO and pH 6.2 conditions, the release of dissolved As from the upper sediment might be primarily controlled by the acidic dissolution, oxidation of arsenic sulfides, and reduction in MnOOH. In deeper sediment layers, the release of As might be predominantly influenced by the reduction dissolution of Fe/Mn-OOH due to the weakly reduced environment.

Under low DO conditions, in the initial stage of cultivation with pH 6.2 (0–6 days), both acidic dissolution and reduction reactions of Fe/Mn-OOH might occur simultaneously at the sediment–water interface, resulting in significantly higher concentrations of dissolved As(V) + As(III), Fe, and Mn compared to other pH conditions (*p* < 0.05). As the pH at the sediment–water interface buffered to weakly neutral conditions, the effect of acidic dissolution decreased, and the increase in dissolved As at the sediment–water interface became more related to the anaerobic reduction in Fe/Mn-OOH and sulfate. The concentrations of dissolved As(V) + As(III), Fe, Mn, and S in the pore water all increased synchronously, reaching 20.36 μg/L on the 17th day. By the 30th day of cultivation, the average concentration of dissolved As(V) + As(III) in the pore water continued to increase to 49.46 μg/L, instead of decreasing synchronously with dissolved Fe, Mn, and S. This suggested that reduction reactions of As might be occurring as it has been shown that As could be reduced at Eh values below +100 mV [45]. On the 30th day of low DO and pH 6.2 cultivation, the Eh below 3 cm at the sediment–water interface was <+100 mV, and the average As(III)/As(V) ratio increased to 4.47, indicating a close relationship between the increase in dissolved As(V) + As(III) and As reduction reactions.

Under high DO conditions, in the initial stage of cultivation with an initial pH of 9.5 (0–6 days), the pH of the water was in a weakly alkaline/alkaline range. At the sediment–water interface (0 to −2 cm), the concentration of dissolved As(V) + As(III) significantly increased to 18.14 μg/L, which was 1.66 times higher than the concentration on the first day. However, no synchronous significant increases in dissolved Fe and Mn concentrations were observed at this location (Figure 3 and Figure 4). Therefore, during this stage, the release of As might not be primarily controlled by the reduction dissolution of Fe/Mn-OOH.

**Figure 7:**
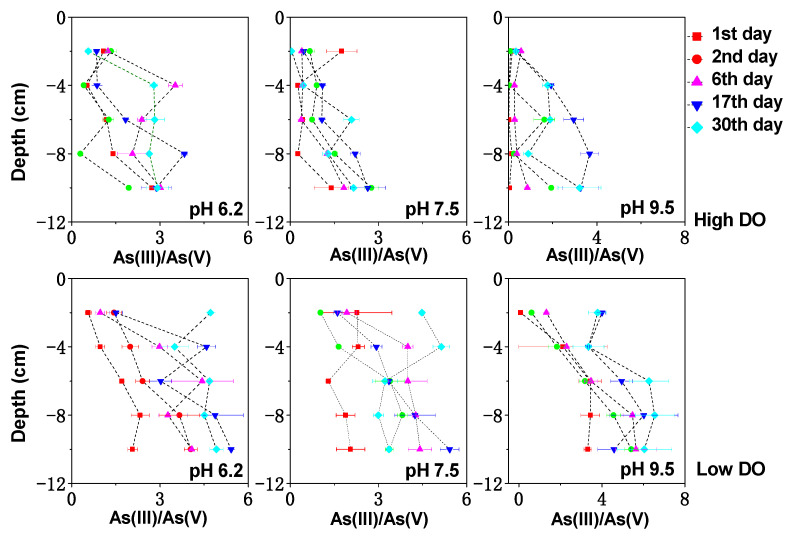
The effect of initial pH on As(III)/As(V) in pore water under different DO conditions.

From Figure 7, it can be seen that the ratio of As(III)/As(V) in the dissolved As in the upper pore water during the initial cultivation period ranges from 0.12 to 0.42, with a considerable proportion of As(V). Due to the presence of OH- ions in the water during this stage, they could compete with arsenate ions for adsorption sites and promote the desorption of arsenate [46,47]. Therefore, the release of dissolved As during this stage is likely mainly attributed to ion competition desorption. In addition, the oxidation of arsenic sulfides was also an important source of As during this stage. Studies have shown that under higher pH conditions, more As could be released through the oxidation of arsenic sulfides [48]. The decrease in the concentration of dissolved S in the pore water at the sediment–water interface during the initial 6 days of cultivation, from 0.046 mg/L to 0.039 mg/L, indicated a correlation between the oxidation of arsenic sulfides and the increase in As concentration.

**Figure 8:**
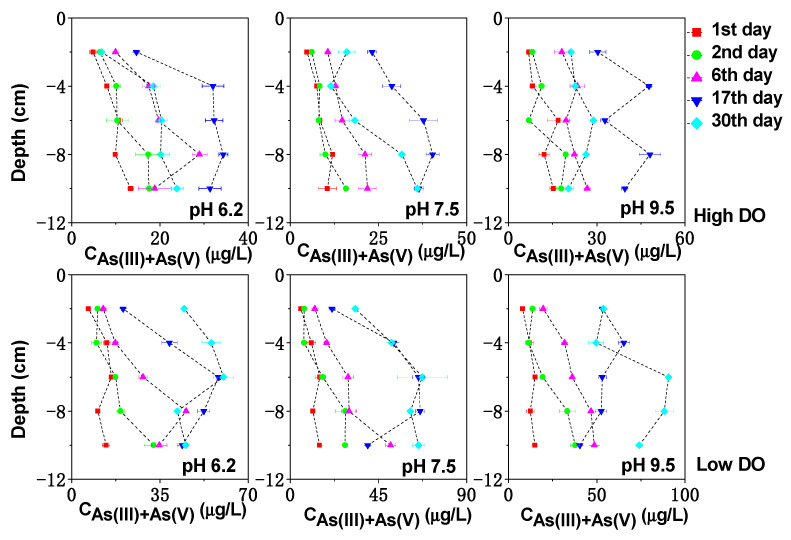
The effect of initial pH on As(III) + As(V) in pore water under different DO conditions.

By the 17th day of cultivation, as the pH of the water buffered to weakly alkaline conditions (Figure 2), ion exchange still occurred at the sediment–water interface and the reduction dissolution of Fe/Mn-OOH in the deeper sediment layers allowed for the continuous upward migration of As. Consequently, the concentration of dissolved As(V) + As(III) at the sediment–water interface continued to increase to 30.37 μg/L (Figure 8), with an average concentration peaking at 39.72 μg/L. Starting from the 17th day of cultivation, the ratio of As(III)/As(V) increased in the dissolved As in the deeper pore water (Figure 7), which might be related to the release of dissolved Mn in the reduced environment of the deeper sediment layers. Studies have shown that Mn can react with As(V) to form Mn_3_(AsO4)_2_ precipitates [49], leading to a decrease in As(V) concentration. By the 30th day of cultivation, as the Eh increases again, the concentrations of dissolved As, Fe, and Mn in the pore water decreased simultaneously, indicating the potential occurrence of co-precipitation and co-adsorption processes. Therefore, under pH 9.5 cultivation conditions, the release of dissolved As at the sediment–water interface was mainly controlled by the combined effects of ion exchange and oxidation of arsenic sulfide, with the reduction dissolution of Fe/Mn-OOH in the deeper sediment layers playing a more dominant role.

Under low DO and pH 9.5 conditions, the microbial activities related to As transformation, such as arsenate-reducing bacteria, iron-reducing bacteria, and sulfate-reducing bacteria, were generally less active under alkaline conditions [50]. Therefore, during the initial 0–6 days of low DO and pH 9.5 cultivation, the release of As was mainly governed by chemical reactions such as ion exchange and the chemical reduction in Fe/Mn-OOH. By the 17th day, when the pH of the water decreased to weakly alkaline conditions, microbial activities gradually recovered. With the sediment in a stronger anaerobic environment (Figure 1), there was an increasing trend of arsenate being reduced to arsenite. By the 30th day of cultivation, the concentrations of dissolved As(V) + As(III) and the As(III)/As(V) ratio continued to increase to 71.31 μg/L and 5.20, respectively.

Under high DO and pH 7.5 conditions, the Fe/Mn-OOH at the sediment–water interface was relatively stable and could adsorb or co-precipitate with dissolved As, which is why there was no noticeable change in the concentration of dissolved As(V) + As(III) at the sediment–water interface on the first and second days of cultivation (Figure 8). Although the system was under high DO cultivation, the penetration depth of DO was limited and could not provide sufficient electrons for organic matter decomposition. Due to the abundant organic matter content in the Zhushan Bay sediment, microbial decomposition of organic matter required a large number of electrons. Therefore, the reduction in Fe/Mn-OOH could still occur in the deeper sediment layers when oxygen supply was insufficient. The average concentration of dissolved As(V) + As(III) in the pore water increased from the initial 8.64 μg/L to 33.32 μg/L on the 17th day and then decreased, following a similar trend as the changes in dissolved Fe, Mn, and S in the pore water under the same conditions. Therefore, under high DO and pH 7.5 conditions, the oxidation of arsenic sulfides and the reduction dissolution of Fe/Mn-OOH might be the main sources of dissolved As.

Under low DO and pH 7.5 conditions, the reduction in Fe/Mn-OOH and the reduction in As promoted the release of As. The concentrations of dissolved As(V) + As(III) in the pore water increased continuously from the initial 11.55 μg/L to 55.78 μg/L on the 30th day, which was significantly higher than the As concentration under high DO and pH 7.5 conditions.

During the cultivation process, the average concentration of dissolved As in the pore water showed a certain correlation with dissolved Fe and Mn (Table 2), indicating that the reduction dissolution of sediment Fe/Mn-OOH promoted the release of As, which was consistent with many previous research conclusions [51,52]. Under low DO conditions, due to the strong reduction ability of As itself, the correlation between As and Fe (or Mn) was influenced and was lower than that under high DO conditions. In the low DO cultivation process, the correlation between dissolved As and dissolved S was higher than that under high DO conditions, indicating that the generation of soluble S promoted an increase in dissolved As under low DO conditions. Under low DO conditions, sediments were subject to strong reducing conditions, and sulfates were easily reduced by sulfate-reducing bacteria to form soluble sulfides. During this process, soluble sulfides were likely to combine with ferrous to form iron sulfide complexes (FeS or FeS_2_) [53,54], causing a transformation of strongly adsorbed solid phases of dissolved As and further facilitating the release of As.

The analysis of the potential formation of ferrous sulfide (FeS or FeS_2_) in the pore water under different pH conditions in a low DO environment revealed that the average pK(FeS) and pK(FeS_2_) values in the pore water were smaller than the pKsp (FeS: 17.20; FeS_2_: 22.12) of amorphous ferrous sulfide compounds (Table 3) [55]. Therefore, it was likely that amorphous ferrous sulfide compounds were formed in the pore water during the cultivation process. Since the adsorption capacity of ferrous sulfide compounds for As(III) was relatively low [56,57,58], it contributed to further release of As(III). There was a good linear relationship between pK(FeS) and pK(FeS_2_) and the concentrations of dissolved As(III) + As(V) and the As(III)/As(V) ratio in the pore water (Table 4), indicating that the formation of amorphous ferrous sulfide compounds under anaerobic conditions increased the concentration of As in the pore water and also promoted the transformation of dissolved As species.

### 3.5. Effect of Initial pH on Dissolved As in Overlying Water under Different DO Conditions

**Figure 9:**
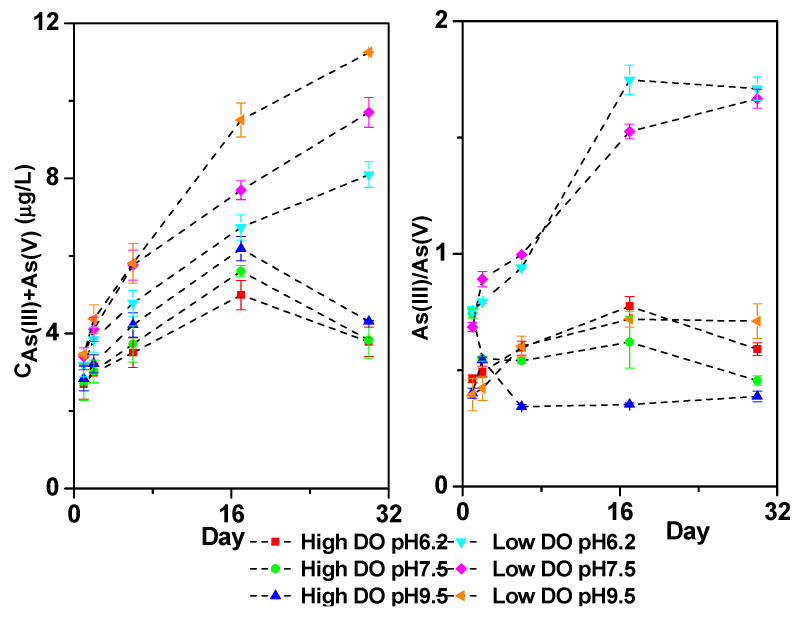
The effect of initial pH of water on As(III) + As(V) concentration and As(III)/As(V) of overlying water under different DO conditions.

Under low DO conditions, the concentration ranges of As(V) + As(III) in the overlying water were 3.19–8.10 μg/L for pH 6.2, 3.41–9.70 μg/L for pH 7.5, and 3.46–11.25 μg/L for pH 9.5, showing a significant increase compared to the As concentration under high DO conditions (Figure 9). The As(III)/As(V) ratio in the overlying water under low DO conditions was relatively high, ranging from 0.76 to 1.75 (pH 6.2), 0.69 to 1.67 (pH 7.5), and 0.39 to 0.72 (pH 9.5). This finding indicates that low DO conditions were more conducive to the migration of As, with a predominant migration in the form of As(III) under initial pH 6.2 and pH 7.5 conditions, while the increase in As concentration in the overlying water under initial pH 9.5 conditions was mainly caused by the migration of As(V).

### 3.6. Cumulative Release Rate of Dissolved As, Fe, Mn, and S in Overlying Water

As shown in Table 5, under high DO cultivation, dissolved Fe and S in the overlying water exhibited adsorption behavior, while dissolved Mn and As increased from 0.013 mg/(m^2^∙d) and 3.61 mg/(m^2^∙d) at initial pH 6.2 to 0.017 mg/(m^2^∙d) and 5.60 mg/(m^2^∙d) at initial pH 9.5, respectively. Under low DO conditions, dissolved As, Fe, Mn, and S in the overlying water were all released, with release rates changing from 0.053, 0.73, 6.31, and 0.38 mg/(m^2^∙d) at pH 6.2 to 0.083, 1.11, 14.27, and 0.83 mg/(m^2^∙d) at pH 9.5. The results indicate that under low DO conditions and initial pH 9.5, the accumulation release rate of each element was the highest, indicating that the redox reactions of Fe, Mn, and S were most intense under this condition, exerting the greatest influence on the upward migration of dissolved As to the overlying water.

### 3.7. Influence of Initial pH of Water on Sediment As Components under Different DO Conditions

**Figure 10:**
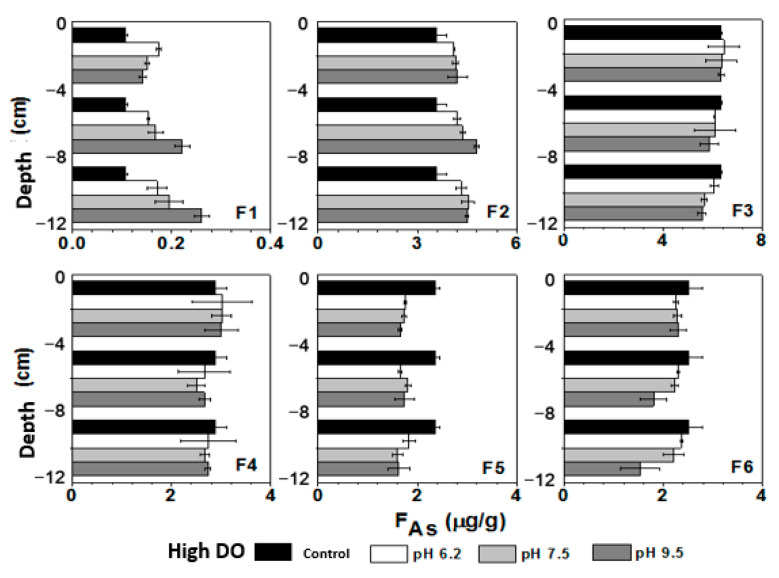

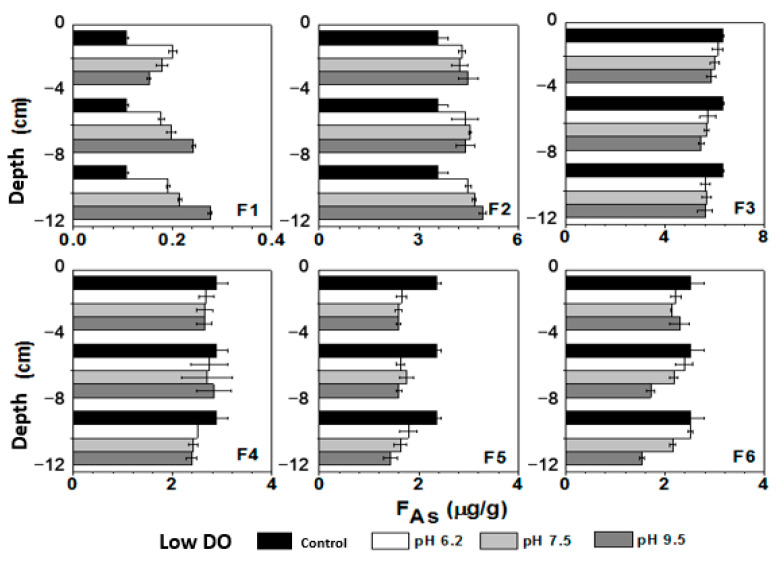
Changes of As components in sediments before and after culture.

The influence of initial pH on As release in different DO conditions needs further analysis by examining the variation in As fractions in sediment. The vertical distribution of As fractions in sediment can be seen in Figure 10. The proportions of exchangeable As, Fe/Mn-OOH-As, and organic matter-bound As in the initial sediment were 20.71%, 51.91%, and 13.29%, respectively. Exchangeable As and Fe/Mn-OOH-As were the dominant forms of As in sediment. Exchangeable As had a high bioavailability and was the most easily released fraction in sediment. Fe/Mn-OOH-As was sensitive to redox conditions, readily undergoing reduction and released under anaerobic conditions, while being prone to oxidation and precipitation under aerobic conditions [59]. Therefore, the geochemical cycling of exchangeable As and Fe/Mn-OOH-As played an important role in the migration of As. Organic matter-bound As was a relatively stable fraction but could slowly release under oxidizing conditions due to the oxidation of organic matter [20], with potential bioavailability.

Under low DO conditions, the average concentration of exchangeable As (weakly exchangeable As + strongly exchangeable As: F1 + F2) in sediment increased from 3.68 μg/g to 4.58 μg/g (pH 6.2), 4.68 μg/g (pH 7.5), and 4.84 μg/g (pH 9.5). The content of exchangeable As increased with depth, indicating a greater conversion of other forms of bound As to exchangeable As with the increase in reduction conditions. Fe/Mn-OOH-As could be divided into amorphous Fe/Mn-OOH-As and crystalline Fe/Mn-OOH-As. The reduction dissolution of amorphous Fe/Mn-OOH-As significantly contributed to the increase in the concentration of dissolved As, with its average content decreasing from the initial 6.34 μg/g to 5.82 μg/g (pH 6.2), 5.77 μg/g (pH 7.5), and 5.64 μg/g (pH 9.5). After the cultivation under initial pH 9.5 conditions, although the overlying water was in a weakly alkaline environment, the low Eh prevented the formation of FeOOH or MnOOH precipitation from dissolved Fe(II) and Mn(II) at the sediment–water interface. As a result, there was also a significant decrease in amorphous Fe/Mn-OOH-As at the sediment–water interface. From Figure 10, it can be seen that the decrease in amorphous Fe/Mn-OOH-As was most pronounced in the deep sediment. In an anaerobic environment, amorphous Fe/Mn-OOH-As underwent reduction dissolution, leading to the dissociation and release of associated As due to the loss of adsorbents. Some of the released As entered the pore water, increasing the concentration of dissolved As. Another portion of As can be re-adsorbed and converted into exchangeable As by the solid phase.

During the increase in reduction conditions, some dissolved As can also be strongly adsorbed and precipitated by secondary iron minerals, leading to the formation of crystalline Fe/Mn-OOH-As [60]. However, from the changes in the content of crystalline Fe/Mn-OOH-As, it can be observed that there was a decrease in crystalline Fe/Mn-OOH-As under different pH conditions after cultivation, indicating that crystalline Fe/Mn-OOH-As also underwent some degree of reduction. The rate of reduction might be greater than the rate of formation, resulting in no increase in crystalline Fe/Mn-OOH-As (Figure 10). Under anaerobic conditions, anaerobic microorganisms can also consume sulfate and organic matter [20,61], converting organic-bound As into inorganic-bound As, significantly promoting As release. From Figure 10, it can be seen that under different pH conditions, the content of organic-bound As decreased from 2.36 μg/g to 1.69 μg/g (pH 6.2), 1.66 μg/g (pH 7.5), and 1.54 μg/g (pH 9.5), respectively.

After cultivation under high DO conditions, there was also an increase in the content of exchangeable As in sediment due to some degree of reduction reactions occurring in the internal environment of the sediment. However, the extent of increase was smaller compared to that under low DO cultivation (Figure 10), with values of 4.38 μg/g (pH 6.2), 4.52 μg/g (pH 7.5), and 4.71 μg/g (pH 9.5). During cultivation, dissolved Fe and Mn in the deep sediment migrated upward and accumulated at the sediment–water interface. After cultivation, the increase in Eh led to the oxidation of dissolved Fe and Mn at the sediment–water interface, forming Fe/Mn-OOH and re-adsorbing dissolved As in the overlying water and pore water at the sediment–water interface. At the sediment–water interface under initial pH 6.2, 7.5, and 9.5 conditions, Fe/Mn-OOH-As increased from 9.22 μg/g to 9.48 μg/g (pH 6.2), 9.36 μg/g (pH 7.5), and 9.33 μg/g (pH 9.5) (Figure 10).

**Figure 11:**
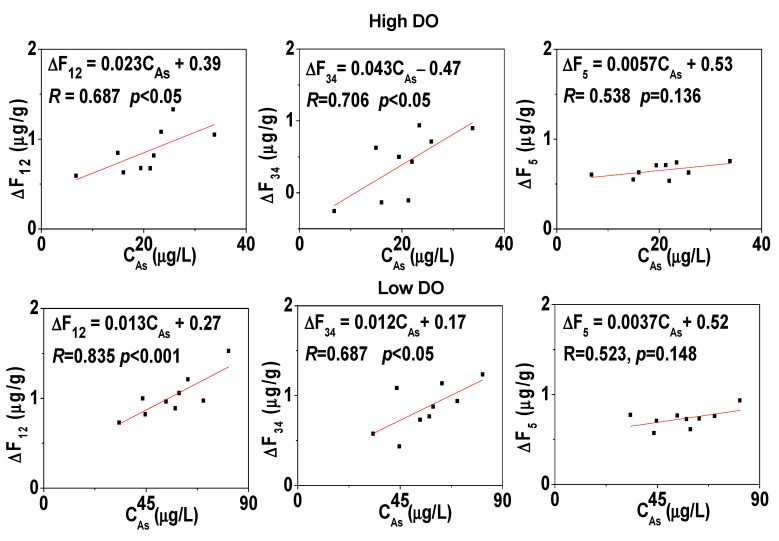
The linear correlation analysis between sediment As component variation and dissolved As in pore water.

After cultivation, the deep sediment still remained under weak reducing conditions, and the changes in various As fractions were similar to those under low DO conditions, with the conversion of Fe/Mn-OOH-As to exchangeable As (Figure 10). The average content of Fe/Mn-OOH-As in sediment decreases to 8.99 μg/g (pH 6.2), 8.76 μg/g (pH 7.5), and 8.71 μg/g (pH 9.5). Under high DO cultivation, there was some degree of oxidation of organic-bound As at the sediment–water interface, while the deep sediment experienced some degree of reduction in organic-bound As, resulting in a decrease in the average content of organic-bound As to 1.74 μg/g (pH 6.2), 1.71 μg/g (pH 7.5), and 1.67 μg/g (pH 9.5). The changes in exchangeable As and Fe/Mn-OOH-As in the sediment were significantly linearly correlated with the concentration of dissolved As in pore water (Figure 11), and there was a certain positive correlation with organic-bound As, indicating that the release of As was closely related to the generation of exchangeable As and the reduction in Fe/Mn-OOH-As, and the changes in organic-bound As also had a certain promoting effect on As release.

## 4. Conclusions

At the initial pH 6.2 condition, the release of dissolved As in sediments was the least, and the next was at the initial 7.5 condition, while at the initial pH 9.5 condition, the competitive adsorption between hydroxyl ions and As was strong, and the release of dissolved As in sediments was the strongest. The strong buffer effect of sediments on the water’s pH made the pH value of the overlying water under different initial pH conditions shift to the neutral direction, and the level of DO became the key factor for the release of sediment As. The release amount and release rate of sediment As under various initial pH conditions in the low DO environment were greater than those in the high DO environment. The dual effects of ion exchange and anaerobic reduction resulted in the strongest release of sediment As at low DO pH 9.5. The formation of amorphous ferrous sulfide compounds under low DO conditions was also an important reason for the increase in As concentration in interstitial water. Sediment exchange As and Fe/Mn-OOH-As were the main sources of dissolved As. The difference in the transfer and transformation of the exchange state As and Fe/Mn-OOH-As produced by different pH and DO conditions led to the obvious difference in the release intensity of the dissolved state As. This study provides a scientific basis to further understand the biogeochemical behavior of arsenic in shallow lakes, strengthen the prevention and control of arsenic pollution risks, and ensure the health of aquatic systems.

## Figures and Tables

**Table 1:** Physical and chemical properties of surface sediments in Lake Taihu.

TAs (mg/kg)	Fe (g/kg)	Mn (g/kg)	FeOOH (g/kg)	MnOOH (g/kg)	TOC (%)	pH	Moisture Content (%)
18.15	35.42	1.41	24.01	0.85	1.44	7.56	52.4

**Table 2:** The correlation analysis between the mean values of dissolved As and dissolved Fe, Mn, and S in pore water.

Fe_HDO_		Mn_HDO_	S_HDO_	Fe_LDO_	Mn_LDO_	S_LDO_
As (pH 6.2)	0.965 **	0.985 **	0.734	0.886 *	0.803	0.875
As (pH 7.5)	0.986 **	0.924 *	0.721	0.942 *	0.790	0.891 *
As (pH 9.5)	0.961 **	0.978 **	0.713	0.882 *	0.869	0.782

HDO: High DO; LDO: Low DO. * and ** indicates a significant relationship at the level *p* < 0.05, *p* < 0.01.

**Table 3:** The range of ionic product constants of amorphous ferrous sulfide (FeS or FeS_2_) in low DO environment at various pH values.

	p*K* (FeS)	p*K* (FeS_2_)
pH 6.2	9.23–10.05	14.37–15.54
pH 7.5	9.14–9.96	14.21–15.37
pH 9.5	8.89–9.89	13.78–15.25

**Table 4:** The linear relationship between *p*K (FeS) and *p*K (FeS_2_) and dissolved *C*_As_ and *R*_As_ (As(III)/As(V)) in pore water under different pH conditions in low DO environment.

	Linear Equation	Linear Equation
pH 6.2	p*K*(FeS)= −0.018 *C*_As_ + 10.14 *r* = −0.892 *	p*K*(FeS_2_)= −0.026 *C*_As_ + 15.70 *r* = −0.894 *
	p*K*(FeS)= −0.26 *R*_As_ + 10.42 *r* = −0.903 *	p*K*(FeS_2_)= −0.37 *R*_As_ + 16.08 *r* = −0.897 *
pH 7.5	p*K*(FeS)= −0.016 *C*_As_ + 10.03 *r* = −0.923 *	p*K*(FeS_2_)= −0.022 *C*_As_ + 15.46 *r* = −0.916 *
	p*K*(FeS)= −0.38 *R*_As_ + 10.69 *r* = −0.903 *	p*K*(FeS_2_)= −0.52 *R*_As_ + 16.38 *r* = −0.898 *
pH 9.5	p*K*(FeS)= −0.015 *C*_As_ + 9.97 *r* = −0.883 *	p*K*(FeS_2_)= −0.022 *C*_As_ + 15.37 *r* = −0.872
	p*K*(FeS)= −0.33 *R*_As_ + 10.65 *r* = −0.917 *	p*K*(FeS_2_)= −0.48 *R*_As_ + 16.35 *r* = −0.908 *

* indicates a significant relationship at the level *p* < 0.05.

**Table 5:** The effect of initial pH of water body on accumulative release rates of As, Fe, Mn, and S of overlying water under different DO conditions.

mg/(m^2^∙d)	pH 6.2		pH 7.5		pH 9.5	
	Low DO	High DO	Low DO	High DO	Low DO	High DO
*r*_Fe_	0.73	−0.47	0.92	−0.42	1.11	−0.50
*r*_Mn_	6.31	3.61	8.99	4.56	14.27	5.60
*r*_S_	0.38	−0.45	0.51	−0.49	0.76	−0.51
*r*_As_	0.053	0.013	0.069	0.014	0.083	0.017

## Data Availability

The data presented in this study are available on request from the corresponding author. The data are not publicly available due to requirements of the research institution.
